# A chromosome-level genome assembly and annotation of the medicinal plant *Lepidium apetalum*

**DOI:** 10.1186/s12863-024-01243-9

**Published:** 2024-06-17

**Authors:** Hang Yan, Yunhao Zhu, Haoyu Jia, Yuanjun Li, Yongguang Han, Xiaoke Zheng, Xiule Yue, Le Zhao, Weisheng Feng

**Affiliations:** 1 School of Pharmacy, Henan University of Chinese Medicine, No. 156 Jinshui East Road, Zhengzhou, Henan, 450046 China; 2 The Engineering and Technology Research Center for Chinese Medicine Development of Henan Province, Zhengzhou, 450046 China; 3 Ministry of Education Key Laboratory of Cell Activities and Stress Adaptations, School of Life Sciences, Lanzhou University, No. 222 Tianshui South Road, Lanzhou, Gansu, 730000 China

**Keywords:** *Lepidium apetalum*, Genome assembly, PacBio sequencing, Hi-C, Transcriptome

## Abstract

**Objectives:**

As a traditional Chinese medicine, *Lepidium apetalum* is commonly used for purging the lung, relieving dyspnea, alleviating edema, and has the significant pharmacological effects on cardiovascular disease, hyperlipidemia, etc. In addition, the seeds of *L. apetalum* are rich in unsaturated fatty acids, sterols, glucosinolates and have a variety of biological activity compounds. To facilitate genomics, phylogenetic and secondary metabolite biosynthesis studies of *L. apetalum*, we assembled the high-resolution genome of *L. apetalum*.

**Data description:**

We completed chromosome-level genome assembly of the *L. apetalum* genome (2n = 32), using Illumina HiSeq and PacBio Sequel sequencing platform as well as high-throughput chromosome conformation capture (Hi-C) technique. The assembled genome was 296.80 Mb in size, 34.41% in GC content, and 23.89% in repeated sequence content, including 316 contigs with a contig N50 of 16.31 Mb. Hi-C scaffolding resulted in 16 chromosomes occupying 99.79% of the assembled genome sequences. A total of 46 584 genes and 105 pseudogenes were predicted, 98.37% of which can be annotated to Nr, GO, KEGG, TrEMBL, SwissPort, Pfam and KOG databases. The high-quality reference genome generated by this study will provide accurate genetic information for the molecular biology research of *L. apetalum*.

## Objective

*Lepidium apetalum* Willd., an annual or biennial herb, belongs to the genus *Lepidium* in the family Brassicaceae and is mainly distributed in the northern part of China [1]. Its dried mature seeds are called “Tinglizi”, which is a traditional Chinese medicine commonly used for purging the lung, relieving dyspnea, and alleviating edema [2], and has the significant pharmacological efficacy for cardiovascular disease, hyperlipidemia, etc [3]. The seeds of *L. apetalum* are rich in fatty oils, cardiac glycosides, glucosinolates and flavonoids etc [4]. . The seeds contain up to 40% fatty oils, of which the unsaturated fatty acid content is as high as 70–91% [5], such as oleic, linoleic, and linolenic acids [6], making *L. apetalum* a potential oilseed crop. In addition, *L. apetalum* is widely distributed in high-altitude alpine region with strong cold resistance, which is an ideal material in the study of cold resistance [7].

Currently, researches on *L. apetalum* mainly focused on pharmacological effects, isolation of new compounds and cold resistance [8], but fewer studies have investigated the key genes involved in secondary metabolites biosynthesis and unsaturated fatty acid accumulation. Advances in molecular biology and gene function studies of *L. apetalum* has been severely limited by the fact that its genome has not been sequenced. Using Illumina short-reads combined with PacBio long-reads and Hi-C technique, we assembled a high-quality chromosome-level reference genome of *L. apetalum*. These results not only provide detailed genetic information for the secondary metabolites biosynthesis and phylogenetic studies of *L. apetalum*, but also lay the foundation for elucidating the molecular mechanism of cold resistance in *L. apetalum*.

### Data description

*L. apetalum* samples were collected from Henan Funiu Mountain National Nature Reserve, Henan Province, China (110°30′E, 32°45′N) and identified by Prof. Chengming Dong of Henan University of Chinese Medicine. The genomic DNA was extracted from *L. apetalum* leaves using a modified CTAB method [9]. Whole genome sequencing of *L. apetalum* was completed by Biomarker Technologies (Beijing, China) utilizing Illumina X Ten platform and PacBio Sequel II platform. The genomic DNA libraries (350 bp) were prepared according to Illumina’s standard protocol, and subjected to paired-end 150 bp (PE 150) sequencing on the Illumina X Ten platform, yielding 30.54 Gb data with the sequencing depth of approximately 101.46 × (Table 1; Data set 1). Illumina sequencing data were analyzed by Jellyfish v2.1.4 and GenomeScope v2.0 to construct K-mer distribution maps with k = 21 for the assessment of *L. apetalum* genome size, GC content, heterozygosity, etc. According to the results of the K-mer analysis, the genome size of *L. apetalum* was about 301.18 Mb, the GC content was 34.14%, the heterozygosity was 0.001%, and the repetitive sequences content was 30.1% (Table 1; Data file 1). Based on the genome survey results, the PacBio library was constructed and circular consensus sequencing (CCS) was performed on PacBio Sequel II platform, which generated 22.12 Gb data (Table 1; Data set 2). Utilizing the HiFi CCS data, the genome sequence was assembled with hifiasm v0.12 [10]. Hi-C fragment libraries (300–700 bp insert length) were constructed as described by Rao [11] and sequenced through Illumina HiSeq X Ten platform, yielding a total of 89.36 Gb data (Table 1; Data set 3). With Hi-C technique assisted genome assembly, the final assembled *L. apetalum* genome was 296.81 Mb in size (2n = 32), consisting of 295 scaffolds, with a scaffold N50 of 17.71 Mb and contig N50 of 16.31 Mb (Table 1; Data files 2–4). The completeness of *L. apetalum* genome assembly was evaluated by BUSCO v5.2.2 with Brassicales database, and complete BUSCO score was 96.67%.

Transcriptome data of different tissues (roots, stems, leaves, seeds) have been deposited in NCBI GenBank under the Bioproject PRJNA1082618 for gene annotation (Table 1; Data set 4). We integrated three methods, homology search, *de novo* prediction, and transcript-based assembly, using EVM v1.1.1 to annotate protein-coding genes in *L. apetalum* genome [12], resulting in 46 584 genes. Finally, a total of 45 825 (98.37%) genes were annotated by searching the Nr, TrEMBL, Pfam, SwissProt, KOG, GO, and KEGG databases (Table 1; Data files 5, 7 and 8). The assembled genome, gene sequences, gene coding sequences (CDS) and annotated proteins of *L. apetalum* were shown in Table 1 (Table 1; Data sets 5 and 6). Repetitive elements constitute 30.1% of the *L. apetalum* genome, including 23.89% transposable elements (TE) and 6.11% tandem repeats. TE sequences were identified and classified by homology search using RepeatMasker v4.10 [13], which resulted in 70.92 Mb TE sequences. Tandem repeats were annotated by MISA v2.1 [14], which eventually yielded 18.13 Mb tandem repeats. Additionally, non-coding RNAs such as 2 392 tRNAs, 2 667 rRNAs, 188 miRNAs, and 105 pseudogenes were annotated. The detailed experimental methodology was described in Data file 6 (Table 1). We collaborated with Prof. Ming Chen of Zhejiang University to integrate the data of the *L. apetalum* genome into the CropGF platform (https://bis.zju.edu.cn/cropgf/), which makes it very convenient to mine and analyze the *L. apetalum* gene family on this platform [15].

**Table 1:** Overview of data files/data sets

Label	Name of data file/data set	File types (file extension)	Data repository and identifier (DOI or accession number)
Data file 1	K-mer analysis for estimating genome size of *L. apetalum*	Image file (.jpg)	Figshare, 10.6084/m9.figshare.25560747.v1 [16]
Data file 2	The assembly statistics of *L. apetalum* genome	Word file (.docx)	Figshare, 10.6084/m9.figshare.25562490.v1 [17]
Data file 3	Heatmap of Hi-C assembly chromosome interactions	Image file (.jpg)	Figshare, 10.6084/m9.figshare.25562910.v1 [18]
Data file 4	Circos plot of *L. apetalum* genome	Image file (.jpg)	Figshare, 10.6084/m9.figshare.25562925.v1 [19]
Data file 5	The statistics of genome annotation	Word file (.docx)	Figshare, 10.6084/m9.figshare.25563267.v1 [20]
Data file 6	The detailed experimental methodology	Word file (.docx)	Figshare, 10.6084/m9.figshare.25569060.v4 [21]
Data file 7	The integrated function annotation of *L. apetalum* genome	Excel file (.xls)	Figshare, 10.6084/m9.figshare.25902172.v1 [22]
Data file 8	Gene function annotation for all transcriptomes	Excel file (.xls)	Figshare, 10.6084/m9.figshare.25902433.v1 [23]
Data set 1	Illumina survey data of *L. apetalum* genome	Fasta files (.fasta)	Identifier, http://identifiers.org/insdc.sra:SRX23808217 [24]
Data set 2	PacBio reads of *L. apetalum* genomic DNA	Fasta files (.fasta)	Identifier, http://identifiers.org/insdc.sra:SRX23808218 [25]
Data set 3	Hi-C reads of *L. apetalum* genomic DNA	Fasta files (.fasta)	Identifier, http://identifiers.org/insdc.sra:SRX24109656 [26]
Data set 4	Transcriptome data of different tissues (stem_young, leaf_young, root, stem_old, seed_young, and leaf_old, respectively)	Fasta files (.fasta)	Identifier, http://identifiers.org/insdc.sra:SRX24178224, http://identifiers.org/insdc.sra:SRX24178223, http://identifiers.org/insdc.sra:SRX24178222, http://identifiers.org/insdc.sra:SRX24178221, http://identifiers.org/insdc.sra:SRX24178220, http://identifiers.org/insdc.sra:SRX24178219 [27]
Data set 5	Genome assembly data for *L. apetalum*	Fasta files (.fasta)	Figshare, 10.6084/m9.figshare.25902229.v2 [28]
Data set 6	Gene CDS and annotated proteins of *L. apetalum*	Fasta files (.fasta)	Figshare, 10.6084/m9.figshare.25913245.v1 [29]

### Limitations

Genome and transcriptome data are available in this study, but there is a lack of proteome and metabolome data from different tissues, as well as multi-omics correlation analysis. There are still 22 gaps in the current version of the *L. apetalum* genome, which can be subsequently filled by ONT’s ultra-long sequencing in combination with existing HiFi CCS data, Hi-C and Illumina data to achieve T2T genome quality.

## Data Availability

The data described in this Data note can be freely and openly accessed on NCBI GenBank under the Bioproject PRJNA1082618, and Figshare with DOIs 10.6084/m9.figshare.25902229.v2 and 10.6084/m9.figshare.25913245.v1, respectively.

## Abbreviations

CDS: Coding Sequence
CTAB: Cetyltrimethylammonium bromide
GO: Gene Ontology
Hi-C: High-throughput chromosome conformation capture
KEGG: Kyoto Encyclopedia of Genes and Genomes
KOG: Eukaryotic Orthologous Groups
Nr: Non-redundant
ONT: Oxford Nanopore Technology
T2T: Telomere-to-Telomere
TE: Transposon Element
